# Semi-Crystalline Copolymer Hydrogels as Smart Drug Carriers: In Vitro Thermo-Responsive Naproxen Release Study

**DOI:** 10.3390/pharmaceutics13020158

**Published:** 2021-01-26

**Authors:** Snežana Ilić-Stojanović, Ljubiša Nikolić, Vesna Nikolić, Slobodan Petrović, Violeta Oro, Žarko Mitić, Stevo Najman

**Affiliations:** 1 Faculty of Technology, University of Niš, Bulevar oslobodjenja 124, 16000 Leskovac, Serbia; nljubisa@tf.ni.ac.rs (L.N.); nikolicvesna@tf.ni.ac.rs (V.N.); 2 Faculty of Technology and Metallurgy, University of Belgrade, 11000 Belgrade, Serbia; sloba@tmf.bg.ac.rs; 3 Department of Plant Diseases, Institute for Plant Protection and Environment, University of Belgrade, 11000 Belgrade, Serbia; viooro@yahoo.com; 4 Faculty of Medicine, University of Niš, Blvd. Dr Zorana Djindjica 81, 18108 Niš, Serbia; zarko.mitic@medfak.ni.ac.rs (Ž.M.); stevo.najman@medfak.ni.ac.rs (S.N.)

**Keywords:** *N*-isopropylacrylamide, 2-hydroxypropyl methacrylate, hydrogels, XRD, drug carrier, modified drug release, naproxen

## Abstract

In this study, poly(*N*-isopropylacrylamide-*co*-2-hydroxypropyl methacrylate) hydrogels were synthesized using free radical initiated copolymerization method. Four hydrogels with different cross-linker concentrations were prepared. Semi-crystalline, cross-linked copolymer networks were confirmed by FTIR, SEM and XRD analysis. Variation of swelling behaviour was monitored gravimetrically and thermo-responsiveness has been noticed. An application of synthesized thermo-responsive hydrogels as carriers for the modulated release of anti-inflammatory model drug was investigated. Moreover, naproxen loading into these hydrogels was also determined using FTIR, SEM and XRD techniques and release was analyzed using HPLC method at simulated physiological conditions. Swelling kinetic and mechanism of water transport, as well as diffusion of naproxen through the hydrogels were analyzed. Thus, the aim of this work was to study various compositions of obtained hydrogels and their possibility of application as a thermo-responsive carrier for prolonged naproxen release in order to evaluate as a potential candidate for drug carrier in future pharmaceutical applications.

## 1. Introduction

Nonsteroidal anti-inflammatory drugs (NSAIDs) are generally used in the symptomatic treatment of rheumatoid arthritis, osteoarthritis, inflammation, acute gout, dysmenorrhoea, metastatic bone pain, headaches, migraines and postoperative pain. They do not cause tolerance and dependence, but the characteristic of their application is the phenomenon of reaching a plateau, i.e., maximum effect that cannot be exceeded by increasing the dose. Most of these drugs exert three types of effects: anti-inflammatory, analgesic and antipyretic effect [1]. Naproxen, (+)-(S)-6-methoxy-alpha-methyl-2-naphthalensacetic acid is a derivative of 2-arylpropionic acid (profene) [2] and is only active in the S-form [3]. It is one of the most potent NSAID which reduces prostaglandin biosynthesis and lowers fever [4]. It is a drug that reduces inflammation and pain in joints and muscles. Peak concentrations in plasma occur within 2–4 h and are somewhat more rapid after the administration of naproxen sodium. However, when it is administrated orally, usefulness is possible for a short period of time, about 8 h [5]. Patients with chronic inflammatory diseases, which require long treatment with NSAIDs, may result complications from long therapies and repeated administrations e.g., gastrointestinal disorder, gastritis, ulcer and bleeding [6].

In order to achieve effective targeted delivery and satisfactory therapeutic effects of naproxen, different polymers were designed as drug carriers. Numerous of investigations outlining ways to improve low solubility of naproxen were published. In the inclusion complexes with β-cyclodextrins, naproxen solubility and release rate were increased [7,8]. Ilić-Stojanović and collaborators developed the inclusion complex naproxen:2-hydroxypropyl-β-cyclodextrin which showed the increased naproxen photostability in a thirty-days period of daylight exposure [9]. The interactions between naproxen, ibuprofen and panadol in the inclusion complexes of α-cyclodextrin, β-cyclodextrin, γ-cyclodextrin and dimethyl-β-cyclodextrin were investigated [10].

Stimuli sensitive polymers are intensively studied for preparation of controlled drug delivery systems for variety of active pharmaceutical ingredients [11,12]. Naproxen solid systems with chitosan, poly(vinylpyrrolidone), as well as ternary systems with hydroxypropyl-β-cyclodextrin and amino acids were also prepared and analyzed [13,14,15]. The inclusion complex of naproxen: β-cyclodextrin (1:1) was directly loaded into pH-sensitive, cross-linked interpolymeric hydrogels based on chitosan and poly(vinyl alcohol) and showed negligible naproxen release in the simulated gastric fluid and sustained release in the intestinal fluid [16]. Wang and collaborators showed that a drug delivery system based on naproxen nanoparticles loaded with chitosan hydrogel has the potential to prevent postoperative abdominal adhesions and to relieve pain, and to contribute to the administration of the hydrophobic drug naproxen [17]. Naproxen agglomerated crystals with hydroxypropylcellulose, obtained by spherical crystallization technique have improved compactness and packaging properties [18]. Molecularly imprinted polymers, using (S)-naproxen as template and the combination of butyl methacrylate and MAA as functional monomers were synthesized with an in situ polymerization reaction and achieved higher chiral resolution and column efficiencies compared to the single monomers [19]. Naproxen particles were designed using porous starch [20]. Nanoparticles containing poly-ε-caprolactone, hydrolyzed polyvinyl alcohol and naproxen, prepared by the solvent emulsification-evaporation method, resulted in naproxen sustained release system, controlled by Fickian diffusion [6]. Eleven formulations corresponding to the different types of naproxen monolithic sustained release matrix tablet were formulated and evaluated to study effect of various grades of matrix forming polymer (hydroxy propyl methyl cellulose) and filler [21]. It was found from Peppas equation that naproxen release follows the non-Fickian mechanism. Structure and hydration properties of hydroxypropyl methylcellulose matrices containing naproxen and naproxen sodium were analyzed and results showed increased chain hydration and stability to drug release by the diffusion mechanism [22]. The poly(ethylene oxide-β-methacrylic acid) micelle, synthesized by atom transfer radical polymerization were applied for the pH-dependent release of naproxen incorporated into the micelle core [23]. This micelle system maintained more than half of naproxen at pH = 1.2 for up to 7 h but was released 100% for 2 h at pH = 8.0. The specific interactions of the hydrogen bond type between naproxen as a model drug and poly(vinylpyrrolidone) and the solubility parameters were analyzed [24,25]. Release study of naproxen from pH sensitive pullulan acetate microsphere provided the pH-sensitive release, i.e., the released amount at pH 7.2 was 75 times more than the one present at the pH 1.2 [26].

Stimuli-responsive hydrogels are materials that can be designed for pharmaceutical application and have an important role in modulated/controlled drug release. They are inert and compatible with the human body tissues and some internal organs [27]. They help solubility increasing, as well as limiting the degradation and toxicity of drugs [1,27]. “Intelligent” hydrogels with elastic properties and soft consistency show similarity to the living tissue. Because of their exceptional properties in biomedical applications and increasing demands for effective treatment, they are considered to be one of the “materials for XXI century”. One of the most widely studied temperature sensitive polymer is poly(*N*-isopropylacrylamide), (*N*iPAm) and its properties were applied to design a series of thermo-sensitive polymeric systems. Interpenetrating polymer networks with *N*-isopropylacrylamide/acrylamide were obtained via in situ free-radical polymerization using synthesized macro-cross-linkers poly(ethylene glycol diacrylate) and poly(ε-caprolactone diacrylate) [28]. The results indicated that the obtained hydrogels had uniform macroporous structures, exhibited rapid swelling/deswelling kinetics and pH sensitivity response. The results of naproxen release showed that this hydrogels exhibited fast release performance. β-Cyclodextrin was modified by maleic anhydride and fixed in the hydrogels network based on *N*-isopropylacrylamide by a series of polymerization reactions. Poly(MAH-β-cyclodextrin-co-NIPAAm) hydrogels demonstrated slow naproxen release efficiency [29]. Property and sustained release effect on sodium naproxen from poly(*N*-isopropylacrylamide-*co*-methacrylamide) compared to the poly(*N*-isopropylacrylamide) showed that release from the copolymer was more sustained than that from the homopolymer. [30]. The influence of the formulation based on *N*-isopropylacrylamide and hydroxypropyl methylcellulose thermosensitive hydrogel for the naproxen sodium release was investigated [31,32,33,34]. Release tests showed that modification of the cross-linker type influenced the properties of synthesized polymeric particles obtained via surfactant free precipitation polymerization [32]. Poly(*N*-isopropylacrylamide-co-poly(g-glutamic acid)-allyl glycidyl ether) hydrogels were obtained by cross-linking polymerization and the morphology, thermal-and pH sensitivity and biodegradability were studied. The hydrogels were loaded with the naproxen and the release studies were performed under simulated physiological conditions [35].

The goals of presented study are application, characterization and evaluation of a series of copolymer poly(*N*-isopropylacrylamide-*co*-2-hydroxypropyl methacrylate), p(*N*iPAm*-co-*HPMet), co-hydrogels, as drug carriers for modulated release of naproxen. The object of the presented study was to provide a naproxen release formulation with modified characteristics resulting in continued levels of the drug above a 24 h period by using thermosensitive properties of synthesized hydrogels. In previous papers, the potential applications of hydrogels for controlled release of caffeine [36], paracetamol [37], and ibuprofen [38], were described. However, similar investigation for modified release of naproxen could not be found in the available literature. The ability of these temperature-responsive hydrogels to exhibit changes in their swelling behavior and pore structure in response to environmental changes make these materials favorable as matrix systems for modified naproxen delivery in human body conditions and are likely to be important in maintaining analgesia. The observed characteristics of the p(*N*iPAm*-co-*HPMet) copolymer indicated that the copolymer could be used as an appropriate carrier for modified drug delivery.

## 2. Materials and Methods

### 2.1. Reagents

*N*-Isopropylacrylamide (*N*iPAm) 99%, 2-hydroxypropyl methacrylate (HPMet) 96.5% and 2,2′-azobis(2-methylpropionitrile) (AZDN) 98% by Acros Organics, Morris Plains, NJ, US; ethylene glycol dimethacylate (EGDM) 97%, methanol Chromasolv for HPLC, 99.9% HPLC grade and naproxen 98.5–100.5% by Fluka Chemical Corp., Buchs, Switzerland; potassium bromide for IR spectroscopy, Potassium bromide (KBr), ≤100%, by Merck KGaA, Darmstadt, DE; acetone by Centrohem, Belgrade, RS; methanol, p.a. by Unichem, Belgrade, RS.

### 2.2. Synthesis of p(NiPAm-co-HPMet) Hydrogels

Cross-linked copolymers consisted of monomer *N*-isopropylacrylamide with 15 mol% of co-monomer 2-hydroxypropyl methacrylate, in relation to the amount of *N*iPAm monomer, were prepared using EGDM for cross-linking and 2.8 mol% of AZDN for the first step of initiation by free radical polymerization method. The cross-linker EGDM concentrations of 0.5, 1.0, 1.5, 2.0 and 3.0 mol% in relation to the total co-monomers mass in the reaction mixtures were employed to observe the effect of cross linker on co-hydrogels. After all reactants diluted in acetone, the homogenized reaction mixture was syringed into glass ampoules, which were then sealed using the heat of the flame while the ampoule was rotating. Sealed ampoules were heated in the temperature regime: 120 min at 70 °C, 60 min at 80 °C, and 30 min at 85 °C until all initiator’s bonds were homolytically cleaved, producing radicals. After cooling, the obtained co-hydrogels were separated from the glass ampoules and cut into smaller cylinders (*d*.*l* = 5.2, where *d* is the diameter, mm, and *l* is the thickness after hydrogel drying, mm). Then, for the purpose of removal of all non-reacted compounds, hydrogels were extracted by methanol during 72 h, after that they were submerged into methanol/distilled water solutions in 75/25, 50/50, 25/75 and 0/100% for a day. Swollen co-hydrogels were dried to constant mass at 40 °C to pass into the xerogel stage for further testing.

### 2.3. Swelling Study

Swelling of co-hydrogels was monitored gravimetrically at the temperature of 5 °C and 38 °C. Four synthesized samples in xerogels states were weighed and then immersed in fluids with simulated physiological pH values 7.4 and 2.0. At predetermined and regular time intervals, the hydrogels were taken out, wiped with filter paper to remove excess fluid, mass were recorded and returned back in the same vial until constant mass was achieved. The swelling ratio, *α_t_*, and equilibrium swelling ratio, *α*_∞_, were according to Equations (1) and (2), respectively:

(1) $$\alpha_{t}=\frac{m_{t}-m_{0}}{m_{0}}$$

(2) $$\alpha_{\infty }=\frac{m_{\infty }-m_{0}}{m_{0}}$$

*m*_0_ is the xerogel mass, *m_t_* is the mass of the swollen hydrogel at the time *t* and *m*_∞_ is the mass of the swollen hydrogel at equilibrium state.

#### 2.3.1. Temperature Responsiveness

The temperature responsiveness of co-hydrogels was monitored within the temperature range of 5–60 °C in a temperature controlled water bath (Sutjeska, Belgrade, RS). The weighted p(*N*iPAm*-co-*HPMet) xerogels were swollen at equilibrium state in distilled water at 5 °C, weighed again and then immersed into the same vials during the temperature increasing to 60 °C. At each particular temperature, hydrogel samples were incubated in distilled water to the equilibrium state for 24 h, wiped with filter paper, weighed *m_t_* and *m*_∞_, and then were put back. The swelling ratio and equilibrium swelling ratio were evaluated using Equations (1) and (2).

#### 2.3.2. Kinetic Analysis of Swelling and Drug Release Processes

In order to analyze the nature of the fluid diffusion process into the co-hydrogels with different cross-linker contents, Fick’s Equation (3) was applied to fit and calculate the experimental data [39,40].

(3) $$\frac{M_{t}}{M_{\infty }}={kt}^{n}$$

*M_t_*/*M*_∞_, is the fractional sorption, *M_t_* is the amount of the absorbed fluid at the time *t*, *M*_∞_ is the amount of the absorbed fluid at the equilibrium state, *n* is the diffusion exponent which is indicative of the transport mechanism, *k* is the kinetic constant characteristic for the polymer network type (min^1/n^).

By taking the logarithm of the Equation (3), the Equation (4) is obtained:

(4) $$\ln F=\ln\;(M_{t}/M_{\infty })=\ln k+n\ln t$$

The values of exponents *n* and *k* were determined from the slope and intercept of the linear relationship between ln*F* and ln*t*. The diffusion coefficient *D* was calculated for the initial phase of swelling to 60% from Equation (5) [41,42,43]:

(5) $$\frac{M_{t}}{M_{\infty }}=\left(\frac{4}{\pi^{0.5}}\right){\left(\frac{D_{t}}{l^{2}}\right)}^{0.5}$$

where *l* is the thickness of the dried sample (cm), and *D* is the diffusion coefficient (cm^2^/min). By taking the logarithm of the Equation (5), the Equation (6) is obtained (the linear relationship between ln(*M_t_/M_e_*) and ln*t*):

(6) $$\ln(\frac{M_{t}}{M_{e}})=\ln(\frac{4D^{1/2}}{\pi^{1/2}l})+\frac{1}{2}\ln t$$

The diffusion coefficient *D* can be calculated from the intercept of the linear relationship between ln(*M_t_*/*M_e_*) and ln*t*, where *t* is the time for which the gel absorbs half the total amount of fluid. Table 1 shows the values of diffusion exponent *n*, which determined the mechanism of water or solvent diffusion [41,42,43].

### 2.4. Naproxen Incorporation into the p(NiPAm-co-HPMet) Hydrogels

In order to study the possibilities of the application of synthesized co-hydrogels as carriers for the modified release of nonsteroidal anti-inflammatory drugs, the naproxen was applied as a model drug. For the purpose of naproxen incorporation into the p(*N*iPAm*-co-*HPMet) polymeric network, synthesized xerogels were swollen in the naproxen standard substance solution, 40 mg/cm^3^ in methanol/distilled water mixture, 80/20, during 48 h at temperature of 5 °C. Naproxen incorporation efficiency (*η*) was calculated using Equation (7):

(7) $$\eta (\%)=\frac{L_{g}}{L_{u}}\times 100$$

where: *L_g_* is the mass of naproxen incorporated into the co-hydrogel (mg/g_xerogel_) and *L_u_* is the maximum of naproxen available mass for incorporation (mg/g_xerogel_).

### 2.5. In Vitro Naproxen Release Study from p(NiPAm-co-HPMet) Hydrogels

The swollen co-hydrogels with loaded naproxen were soaked with 7 cm^3^ of fluids at simulated physiological conditions (pH 2.0 and 7.4 at the temperature of 38 °C). The amount of the released naproxen was monitored for 24 h. Aliquots (100 µL) from the medium with released naproxen were taken in certain intervals of time (after 5 min, 30 min, 60 min, 90 min, 2 h, 3 h, 4 h, 8 h and 24 h), diluted with methanol, filtered through a 0.45 μm cellulose membrane filter and analyzed using HPLC method. The quantification of the amount of the released naproxen as a function of time was performed using the optimized and validated method developed by Somia and colleagues [44]. HPLC Agilent 1100 Series device with a diode-array detector, DAD 1200 Series (Agilent Technologies, Santa Clara, CA, USA), the Purospher^®^ STAR C18, 25 cm × 4.6 mm, 5 µm (Merck KGaA, Darmstadt, Germany) were applied. The mobile phase was methanol:water, 90:10 (*v*/*v*), at pH 2.7 adjusted with phosphoric acid, at the flow rate 1.2 cm^3^/min with isocratic elution. Agilent ChemStation software was used to process the obtained chromatograms.

### 2.6. Characterization of p(NiPAm-co-HPMet) Hydrogel

#### 2.6.1. Fourier Transform Infrared Spectroscopy (FTIR)

Powder samples of synthesized p(*N*iPAm*-co-*HPMet) xerogels, and p(*N*iPAm*-co-*HPMet) with incorporated naproxen were grounded in the Amalgamator (WIG-L-Bug, Dentsply RINN, a Division of Dentsply International Inc., York, PA, USA) to reduce the particle size to less than 5 mm in diameter. FTIR spectra of powder samples (of *N*iPAm*,* naproxen, synthesized p(*N*iPAm*-co-*HPMet) xerogels, and p(*N*iPAm*-co-*HPMet) with incorporated naproxen) with the KBr (about 0.6% related to KBr amount) were subjected to vacuuming and pressing under a pressure of about 200 MPa to form transparent KBr pelets. All FTIR spectra were recorded on a Bomem Hartmann & Braun MB-series FTIR spectrophotometer (Hartmann & Braun, Baptiste, Quebec, QC, Canada) in the wavelength range of 4000–400 cm^−1^. Win-Bomem Easy software was applied for FTIR spectra analysis.

#### 2.6.2. Freeze-Drying

Freeze-drying process of co-hydrogels in equilibrium swollen state before and after incorporation of naproxen was performed on the device LH Leybold, Lyovac GT2 (Frenkendorf, Switzerland). The swollen hydrogels were firstly frozen and after that the amount of fluid was removed by sublimation at −30 °C and 0.05 kPa during 18 h, and in the isothermal desorption phase they were heated at 20 °C at the same pressure. Freeze-dried co-hydrogels were packed under vacuum and stored at 5–7 °C.

#### 2.6.3. Morphology Investigation

Samples of p(*N*iPAm*-co-*HPMet) copolymers in xerogels state and freeze-dried hydrogels in equilibrium swollen state, before and after naproxen incorporation were first metalized with a gold/palladium (85/15) alloy under vacuum using JEOL Fine Coat JFC 1100E Ion Sputter (JEOL Ltd., Tokyo, Japan). After coating, they were scanned on a JEOL Scanning Electron Microscope JSM-5300 (JEOL Ltd., Tokyo, Japan).

#### 2.6.4. X-ray Diffraction Analysis

Powder samples of synthesized p(*N*iPAm*-co-*HPMet) xerogels, naproxen and xerogels with incorporated naproxen were analyzed on Philips PW1030 powder diffractometer (Philips Co., Eindhoven, The Netherlands) by being exposed to monochromatic Cu K_α_ radiation in the range of *2θ* = 7–34° with 0.05° increments and recording time of τ = 5 s. The operating device voltage and the strength of the electric current were 40 kV and 20 mA, respectively.

The crystallinity index (CrI) was determined using the empirical models to evaluate the degree of crystallinity developed by Segal and co-workers [45,46], and Guirguis and Moselhey [47], as a simple way to calculate the relative crystallinity by Equation (8):

(8) $$\mathrm{CrI}=\frac{I_{f}-I_{s}}{I_{f}}\times 100$$

where *I_f_* is the peak intensity of the fundamental (main) band and *I_s_* is the peak intensity of the secondary band. CrI is an empirical measure of relative crystallinity. The variation of the crystallinity index (∆CrI) can be determined using the Equation (9):

(9) $$\Delta \left(\mathrm{CrI}\right)\%=\frac{{\mathrm{CrI}}_{\mathrm{pNIPAM}\text{-}\mathrm{HPMet}\;}-{\mathrm{CrI}}_{\mathrm{pNIPAM}}}{{\mathrm{CrI}}_{\mathrm{pNIPAM}}}\times 100$$

where CrI*_N_*_iPAm*-co-*HPMet_ and CrI*_N_*_iPAm_ are values of crystallinity index for copolymer p(*N*iPAm*-co-*HPMet) and homopolymer p(*N*iPAm), respectively.

### 2.7. Statistical Analysis

The swelling study, XRD and in vitro naproxen release study were carried out in triplicate. Standard deviation of mean of triplicate data from the individual data was quantified using Origin software, for statistical analysis. The statistical analysis was carried out by one-way analysis of variance (ANOVA) and *p* values < 0.05 were considered to be statistically significant.

## 3. Results

### 3.1. Synthesis of p(NiPAM-co-HPMet) Hydrogel

The synthesis of p(*N*iPAM*-co-*HPMet) hydrogels was performed with 15 mol% of co-monomer HPMet and 0.5, 1.0, 1.5, 2.0 and 3.0 mol% of EGDM. The obtained product with 0.5 mol% of EGDM cross-linker remained in liquid consistency after polymerization without required hydrogel consistency, and it was not used for further analysis.

### 3.2. Swelling of p(NiPAm-co-HPMet) Hydrogels

The behaviour of p(*N*iPAm-*co*-HPMet) xerogel samples during swelling in distilled water was monitored at range of 5 °C–60 °C. The swelling ratio, α_t_, was calculated according to Equation (3), and the equilibrium swelling ratio, *α*_∞_, according to Equation (4). The mass of xerogel samples (m_°_) was measured until equilibrium was established (m_∞_).

#### 3.2.1. Equilibrium Swelling

**Figure 1:**
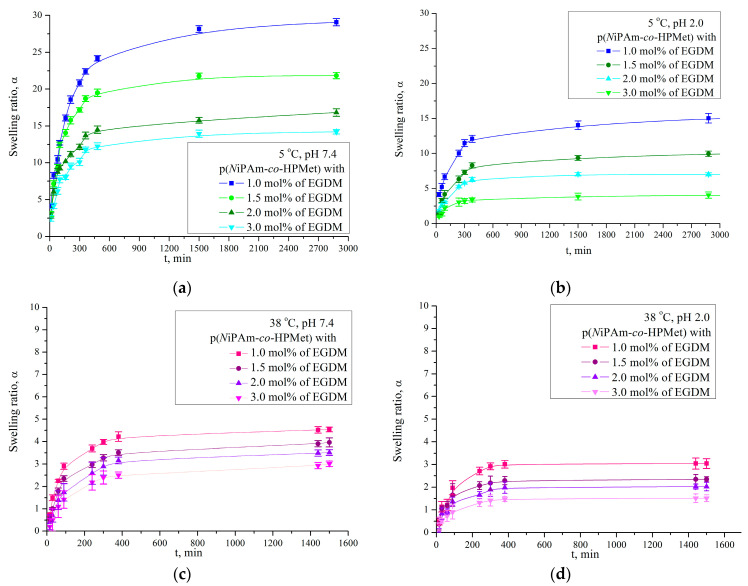
Change in the swelling ratio, α, of p(*N*iPAm-*co*-HPMet) with 15 mol% HPMet and 1.0; 1.5; 2.0 and 3.0 mol% of EGDM, depending on temperature at: (**a**) 5 °C and pH 7.4; (**b**) 5 °C and pH 2.0, (**c**) 38 °C and pH 2.0; (**d**) 38 °C and pH 2.0. In each figure, error bars represent the standard deviation of three replicates.

In order to determine isothermal swelling behavior a series of p(*N*iPAm-*co*-HPMet) hydrogels with different cross-linker content (1.0, 1.5, 2.0 and 3.0 mol% of EGDM) at different pH values (2.0 and 7.4) and temperatures (5 °C and 38 °C) (Figure 1) was monitored.

The swelling capacity of a series of co-hydrogels depends on the temperature of swelling medium and gradually decreases when the cross-linker amount increases (1.0, 1.5, 2.0, and 3.0 mol% of EGDM). All analyzed samples in distilled water increasingly swelled from the initial moment to the first 400 min of swelling at both temperatures.

At 5 °C co-hydrogels swelled gradually until equilibrium swelling was reached after 2 days at *α*_∞_ = 29.07 for sample with lesser cross-linker amount (1.0 mol% of EGDM) (Figure 1a). It can be noted that the equilibrium swelling ratio at a lower temperature is higher than the achieved value at 20 °C in the literature for co-hydrogels (*α*_∞_ = 21.46) [48]. This lower temperature could be applied as a solution for better active ingredient incorporation.

The co-hydrogels swell significantly lesser at 38 °C and reach equilibrium state after 400 min, i.e., 1 g of hydrogel with 1 mol% EGDM can absorb nearly 4.56 g of water from the sample with 1 mol% of EGDM (Figure 1b). The achieved swelling capacities are slightly higher in relation to the values at 40 °C (*α*_∞_ = 4.04 hydrogel with 1.0 mol% of EGDM) presented in previous papers [37,48]. In both temperatures (5 °C and 38 °C) co-hydrogels had the slightly higher values of the equilibrium swelling ratio in the alkaline medium at pH 7.4 (29.077 and 4.541) than in the acidic medium at pH 2.0 (15.411 and 3.044) similar to the published data [36,37].

The analysis of obtained results showed that the swelling ratio decreases with increasing EGDM content, due to many available cross-linker’s molecules that form a polymer network with a lot of cross-linking places. The small hole free volume in copolymer network with a higher cross-linker content reduces the possibility of swelling and an equilibrium swelling ratio was achieved in a shorter time. In contrast, when the EGDM cross-linker’s amount is lesser, long-chain structures have fewer cross-linking places at the same distance, causing the polymer network pore to expand more and thus increase the water absorption capacity. According to free volume theory, the total free volume could be divided into interstitial free volume (which is not implicated in facilitating transport through the liquid) and the hole-free volume, and self-diffusivity of penetrant could be easily predicted [49]. It is presumed that the hole free volume dictate molecular transport and can be predicted according to the type of absorbed fluid.

The achieved swelling ratio for co-hydrogels synthesized by gamma radiation method have a lesser value compared to hydrogels obtained by free radical polymerization [36]. The cross-linking degree of copolymer depends on the intensity of gamma rays during polymerization, and hydrogel p(*N*iPAm-*co*-HPMet) with 17% of HPMet obtained with an applied γ-ray dose of 20 kGy in water has a swelling rate of about 5.5. A similar result with equilibrium swelling ratio of about 5.0 was reached for hydrogel p(*N*iPAm-*co*-HPMet) with 10 wt% of HPMet, obtained by radical polymerization in aqueous solution with 2% of *N*,*N*-methylene-bis(acrylamide) as a cross-linker [50].

#### 3.2.2. Swelling Kinetics

Values of equilibrium swelling ratio (*α*_∞_) and swelling kinetic parameters for synthesized co-hydrogels: diffusion exponent (*n*), kinetic constant proportionality (*k*) diffusion coefficient (*D*), and the correlation coefficient (R^2^), determined by using Equations (3)–(6), at 5 °C and 38 °C at pH values 7.4 and 2.0 are shown in Table 2, respectively.

The values of the diffusion exponents, *n*, indicate that the fluid transport mechanism of co-hydrogels in fluid with pH 7.4 corresponds to “Less Fickian” diffusion at both temperatures (5 °C and 38 °C) for all samples, i.e., the solvent transport in the polymeric network is considerably slower than the relaxation of polymer chains [51]. The process of diffusion is influenced by the presence of “microcavities”, already existed in the polymer network structure [52,53,54]. Only the diffusion exponent for the sample with 1.5 mol% of EGDM at 5 °C and pH 7.4 and all samples in acidic fluid (pH 2.0) showed anomaly in the mechanism of water transport, i.e., the swelling process is controlled by water diffusion and relaxation of polymer chains. Similar values for the diffusion exponent for hydrogels p(*N*iPAm*-co-*HPMet) at 25 °C have been reported in the literature [36,38]. The values of the kinetic constant, *k*, are higher at 38 °C due to the reduction of the hole free volume in the polymer network [37]. The values of the diffusion coefficient, *D*, at 5 °C are in range of 0.326–5.379 × 10^−6^ cm^2^/min, while at 38 °C, above lower critical solution temperature (LCST), they are in the range of 7.066 × 10^−5^–1.899 × 10^−4^ cm^2^/min, indicating faster diffusion, which is similar to the results published in the literature [55,56,57].

#### 3.2.3. Temperature Responsiveness

The influence of temperature to the swelling properties of synthesized p(*N*iPAm-*co*-HPMet) hydrogels, was monitored throughout the change in the swelling ratio at the temperature increasing from 5 °C to 60 °C in distilled water in order to test their temperature responsivity.

**Figure 2:**
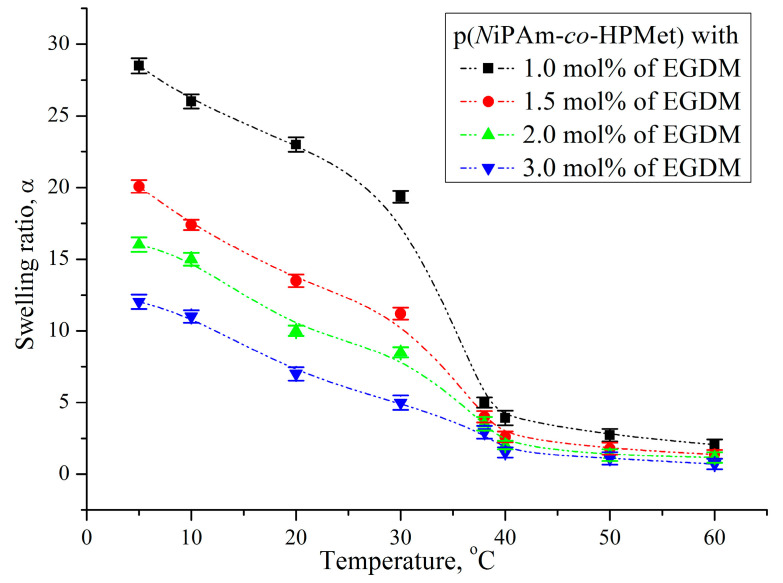
Change in the swelling ratio, α, of p(*N*iPAm-*co*-HPMet) depending on temperature. Error bars represent the standard deviation of three replicates.

All analyzed co-hydrogels show a “smart” reaction which is reflected in a significant decrease in the swelling ratio with increasing external temperature, which can be seen in Figure 2. The co-hydrogels achieved the most intense volume contraction, i.e., they exhibit volume phase transition temperature (VPTT) after temperature increase in the range of 30–38 °C which classifies them as negative thermo-sensitive (LCST). Water retention capacity continuously decreases with increasing amount of EGDM in the hydrogel sample. This behaviour was expected because a higher content of cross-linker increases the density of the polymer network, reducing the elasticity. The most pronounced thermo-sensitivity was shown by the sample of p(*N*iPAm-*co*-HPMet) with 1.0 mol% EGDM, and the sample with 3 mol% EGDM showed a very small change in the degree of swelling and phase transition. The highest values of swelling capacity for all samples of co-hydrogels were achieved at 5 °C, when e.g., 1 g of a sample with 1.0 mol% of EGDM absorbed 29.08 g of water. As the temperature increased to 60 °C, the hydrogels dehydrated with the release of free, unbound water from the polymer network, so that 1 g of the same sample retained only 2.06 g of water. This behavior of hydrogels showed similarity with the results previously published in the literature [36,38,47,58]. In the swollen state at lower temperatures, intermolecular hydrogen bonds between the side groups of hydrogels (-OH and -NH) with water molecules are rewarded. They are stronger than intramolecular interactions of the main chains and prevent its aggregation. With temperature increasing above the LCST the intermolecular hydrogen bonds break, because long polymer C-C chains tend to reduce their surface area and form intramolecular hydrogen bonds between the side -OH groups of HPMet and electronegative oxygen atoms from the ester functional group (HPMet and EGDM). or with electronegative nitrogen from the -NH group of NIPAM. They become dominant over intermolecular hydrogen bonds and lead to dehydration of the thermosensitive hydrogel [59,60]. This “smart” response of co-hydrogels described as negative thermo-sensitivity was applied to absorb pharmaceutical active ingredient from aqueous fluid at a lower temperature and to study their release when temperature increases above LCST, similar to human body temperature in fever state.

### 3.3. FTIR Analysis

#### 3.3.1. FTIR Analysis of p(*N*iPAm-co-HPMet) Hydrogels

**Figure 3:**
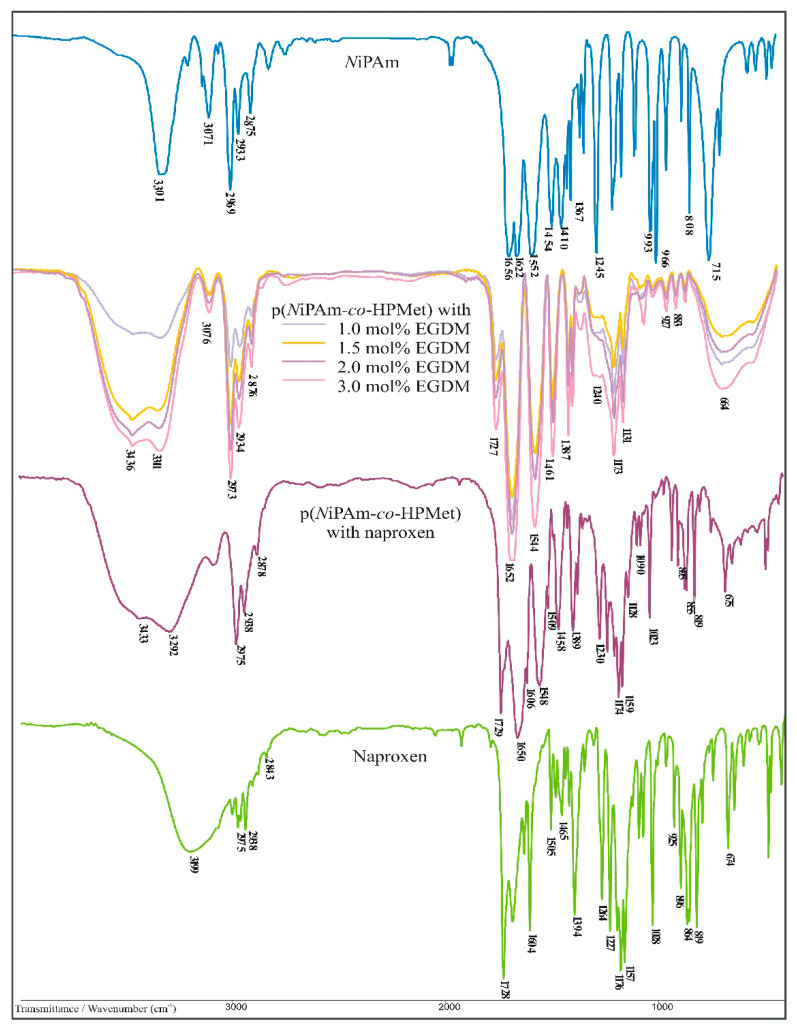
FTIR spectra of monomer *N*iPAm, synthesized p(*N*iPAm*-co-*HPMet) hydrogels with 1.0, 1.5, 2.0 and 3.0 mol% of EGDM, p(*N*iPAm*-co-*HPMet) hydrogel with 1.0 mol% of EGDM with incorporated naproxen and naproxen.

FTIR spectra of monomer *N*iPAm, synthesized p(*N*iPAm*-co-*HPMet) hydrogels with 1.0, 1.5, 2.0 and 3.0 mol% of EGDM are presented in Figure 3. When compared a series of four FTIR spectra from synthesized copolymers p(*N*iPAm*-co-*HPMet) with the FTIR spectrum of *N*iPAm monomer, some differences can be noticed, which indicates formation of new structures, i.e., some bands are not present in the spectrum of *N*iPAm monomer, absence of several bands characteristic for monomers, as well as the change of intensity and position of certain bands. In all FTIR spectra of the p(*N*iPAm*-co-*HPMet) there are no absorption bands at 1620–1640 cm^−1^, which originate from the stretching vibration of C = C double bond and confirm that the polymerization reaction is performed via breaking double bonds of vinyl groups from monomers and cross-linkers. The intensity of the bands originating from the stretching vibrations of vinyl group = C-H (maximum in the monomer FTIR spectrum at 3071 cm^−1^) significantly decrease and shift by 5 units to lower wavenumbers, confirming that polymerization occurred by breaking the double bond of vinyl groups [61]. The presence of some residual amount of unreacted monomer *N*iPAM trapped into the polymer network can be noticed. In all FTIR spectra of copolymers, a broad bands in the range from 3600 cm^−1^ to 3100 cm^−1^ were observed, where the first bands at 3311 cm^−1^ originate from the stretching vibrations of side N-H bonds, ν(NH), with maxima shifted by 10 units to lower wavenumbers than in the spectrum of monomer. Second band maxima at 3436 cm^−1^ were the results of the stretching vibrations of O-H bonds, ν(OH), from co-monomer HPMet present in the side-chains of polymer network. In the p(*N*iPAm*-co-*HPMet) spectra the maxima intensities of amide bands I, ν (C=O), were shifted by 4 units to higher wavenumbers (1652 cm^−1^) and amide bands II, δ(N-H), of amide bands II were shifted by 2 unit to lower wavenumbers (1554 cm^−1^), indicating that C=O and N-H bonds participated in the formation of intramolecular hydrogen bonds. The presence of the bands originating from stretching vibrations of C-H bond of methyl and methylene groups in the range of 2800 to 3000 cm ^−1^, as well as the stretching vibrations of keto groups C=O from ester, ν (C=O), at 1729 cm^−1^ were noticed in all spectra. Individually divergences in absorption bands intensity in the FTIR spectra of series p(*N*iPAm*-co-*HPMet) copolymers compared to the monomer *N*iPAM are a consequence of the polymerization and crosslinking reaction during the formation of hydrogel. FTIR spectra a series of synthesized p(*N*iPAm*-co-*HPMet) copolymers are matching with the published data [36,61].

#### 3.3.2. FTIR Analysis of p(*N*iPAm-co-HPMet) Hydrogels with Incorporated Naproxen

The FTIR spectra of p(*N*iPAm-*co-*HPMet), sample with 1.0 mol% of EGDM, with incorporated naproxen and naproxen are shown in Figure 3 and the maxima of the characteristic absorption bands before and after naproxen incorporation are given in Table 3. Shifts in the position of individual bands in this spectrum indicate some interactions of naproxen with the p(*N*iPAm-*co-*HPMet) copolymer. Since free -OH groups, amide groups and ester groups are present in the structure of this hydrogels, they may be important for non-covalent interactions with naproxen, especially with the ether, C-O and carboxyl groups, i.e., via the carbonyl C=O and -OH group. For this reason, changes in the maxima position of these functional groups’ bands were monitored. The band of stretching vibrations of the -OH group from the p(*N*iPAm-*co-*HPMet) in the spectrum of this copolymer with incorporated naproxen exists and it is shifted by 3 units to lower values of wavenumbers (3433 cm^−1^), which means that it participated in the formation of non-covalent interaction. Additionally, maxima displacements of the bands from the naproxen‘s -OH group, from in-plane and out-of-plane bending vibrations were observed in the spectrum of copolymers with naproxen. The in-plane bending vibrations, δ(OH) absorbs at 1389 cm^−1^, are shifted by 5 units, while out-of-plane bending vibrations δ(OH) are shifted by 1 unit (925 cm^−1^) both towards lower wavenumbers, which confirms their participation in the interaction with the copolymer. Additionally, the shifts in the stretching vibrations band of the C=O group from the ester (1729 cm^−1^), towards higher wavenumbers for 1 unit relative to the spectra of the copolymers and for 2 units relative to the spectra of naproxen were observed. The N-H group stretching vibration band from the copolymer side-chains in the spectrum of copolymers with naproxen was shifted by 19 units (3292 cm^-1^) to lower wave numbers also indicating the interaction with naproxen. The maximum of amide band I in the spectrum of copolymers with naproxen occurs at 1650 cm^−1^ and is shifted by 2 units to lower wavenumbers of copolymers, while the maximum of amide band II is shifted by 8 units to higher wavenumbers and occurs at 1552 cm^−1^. The band of stretching vibrations of C-O group from the ether‘s part of the naproxen structure was shifted by 3 units to higher wavenumbers in the spectrum of copolymers with naproxen, occurring at 1230 cm^−1^, indicating that it interacted with free -NH or secondary -OH groups from side chains of copolymers by the hydrogen bond formation. Analysis of the FTIR spectra indicates that hydrogen bonds were formed between the naproxen and the copolymers via the carboxyl group, i.e., -OH and C=O and ether C-O groups of naproxen and -OH and -NH groups in the copolymer side chains.

### 3.4. XRD Analysis of p(NiPAm-co-HPMet) Hydrogels after Synthesis and with Incorporated Naproxen

**Figure 4:**
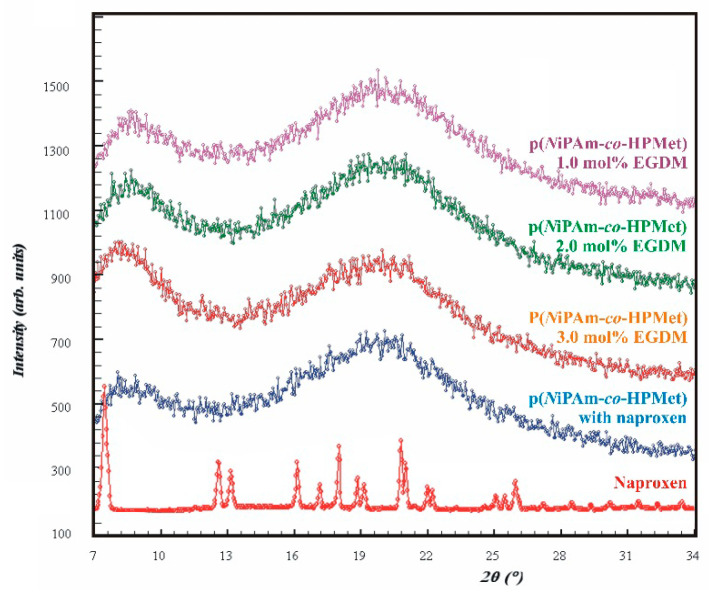
XRD diffractograms of: the copolymers p(*N*iPAm-co-HPMet) with 1.0 mol%, 2.0 mol% and 3.0 mol% of cross-linker EGDM; p(*N*iPAm-co-HPMet) with 1.0 mol% of EGDM with incorporated naproxen; and naproxen.

Three XRD diffractograms samples of synthesized p(*N*iPAm*-co-*HPMet) copolymers with 1.0, 2.0 and 3.0 mol% of EGDM obtained by X-ray diffraction analyses were presentetd in Figure 4.

All of three XRD patterns of p(*N*iPAm-*co*-HPMet) copolymers samples contain two broad, unstructured diffraction peaks with low intensity and indicate a low degree of crystallinity, a small crystallite size and dislocations of the crystal structure at large distances. The first peaks (s) are narrower and occur at diffraction angle values around 8°. The second, broader peaks, named fundamental (f), occur at a diffraction angle of about 20°, which is consistent with published data [58,61]. Diffractograms of all samples show no clearly defined reflections, which means they do not have the orderly crystalline structure, but they are semicrystalline, i.e., amorphous-crystalline. The authors of this study compared obtained results with the diffractograms of p(*N*iPAM) homopolymer, synthesized using free radical polymerization method, analog as described, which was identical as previously published [61]. All of three XRD patterns of copolymers p(*N*iPAm-*co*-HPMet) presented a high similarity with XRD pattern of homopolymer p(*N*iPAM). The value of diffraction intensity for fundamental (I_f_) and secondary (I_s_) peaks from and diffraction angle values of p(*N*iPAm-*co*-HPMet) and p(*N*iPAM) hydrogels were presented in Table 4.

XRD diffractograms of naproxen and p(*N*iPAm-co-HPMet) hydrogel sample with 1.0 mol% of EGDM after naproxen incorporation were also presented in Figure 4. XRD pattern of naproxen with well-structured sharp peaks and clearly defined reflections confirmed its crystalline structure.

In the XRD diffractogram of co-hydrogel with incorporated naproxen (Figure 4) absence of any peaks originating from naproxen, only XRD pattern similar to pure co-hydrogels before naproxen incorporation can be observed [16]. This result indicates that naproxen was efficaciously incorporated into the network of co-hydrogel. The value of diffraction intensity for fundamental peaks (I_f_) and secondary (I_s_) peaks from XRD patterns and diffraction angle values for the co-hydrogel after naproxen incorporation were presented in Table 4.

Diffraction lines of p(*N*iPAm-*co*-HPMet) samples show two maxima of the reflection. In the samples with reduced EGDM content the intensity of secondary peaks (I_s_) increases (Table 4). This is the result of defects formation in the structure which causes the crystallite size decrease and the amorphousness increase. This gives the appearance of the diffraction lines widening. XRD analysis shows that the presence of HPMet monomers in the copolymer structure of p(*N*iPAm-*co*-HPMet) reduces crystallinity and intensifies the amorphousness in comparison to XRD diffractogram of p(*N*iPAM) homopolymer (Figure 4).

X-ray diffraction analysis is most frequently used to measure the crystallinity in the polymers. Two simple empirical models to evaluate relative crystallinity of co-hydrogels were applied. The X-ray crystallinity index (CrI) was determined by the Equation (8) and the variation in the crystallinity index (∆CrI) was calculated by the Equation (9). The obtained values of CrI and ∆CrI for the synthesized co-hydrogels samples are given in Table 5 and show compliance with literature data for similar hydrogels [58,61]. The lower values of the crystallinity index and negative values of the variation in the crystallinity index (ΔCrI) for the p(NiPAm-co-HPMet) compared to the values of homopolymer p(NiPAM) indicate structural irregularity and reduced crystallinity. The existence of dislocations, as high-density crystalline defects, causes a diffraction peak to broaden. The crystallinity reduction is the consequence of alteration in structure of side chains because of the presence of HPMet comonomer. The OH groups from co-monomer HPMet lead to changes of the structure and physical-chemical properties of the polymer network, e.g., possibility to form intermolecular hydrogen bonds. The increase of co-hydrogel crystallinity as a result of the increasing amount of cross-linker EGDM from 1 to 3 mol% can be observed. After naproxen incorporation, the crystallinity index (CrI) and the variation of crystallinity index (ΔCrI) were slightly increased. XRD analysis gave a valuable information on the crystal structure of p(*N*iPAm-*co*-HPMet) copolymers before and after naproxen incorporation. The secondary (I_s_) peak was shifted in the diffractogram of co-hydrogel with incorporated naproxen. This is probably result of hydrogen bonds formed between the naproxen and the co-hydrogel, and it is in correlation with analysis of FTIR spectra.

### 3.5. Morphology Analysis

#### 3.5.1. Morphology Investigation of p(*N*iPAm-co-HPMet) after Synthesis

**Figure 5:**
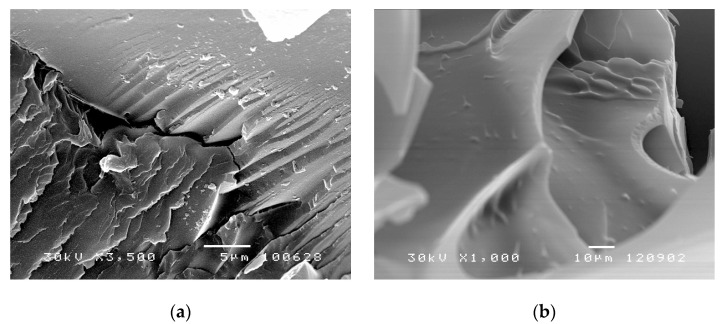
SEM micrographs of p(*NiPAm-co-HPMet*) copolymers: (**a**) in xerogel state with 1.0 mol% of EGDM, (magnification 3500×, scale bar 5 μm) and (**b**) freeze-dried hydrogels swollen to equilibrium state with 3.0 mol% of EGDM (magnification 1000×, scale bar 10 μm).

Morphology of synthesized p(*N*iPAm-*co*-HPMet) copolymers in xerogel state were given in SEM micrographs in Figure 5a, and morphology of freeze-dried hydrogel previously swollen to equilibrium state were given in SEM micrographs in Figure 5b.

The SEM micrographs of synthesized p(*N*iPAm-*co*-HPMet) hydrogels reveals porous nature of hydrogel network. The presence of pores in the co-hydrogel structure facilitates the water passing through the hydrogel network, which is especially important in hydrogels application as drug carriers. It can be estimated that the pores in the xerogel state are on average about 30 μm and bigger than 70 μm in swollen state. Additionally, the alternation of semi-ordered/crystalline and amorphous regions are observed, which is probably due to the influence of the amount of cross-linker on the size and distribution of pores. Cracks of up to more than 5 μm are also observed as well as the appearance of more regulated areas, which also reflected on the swelling ability.

#### 3.5.2. Morphology Investigation of p(*N*iPAm-co-HPMet) with Incorporated Naproxen

**Figure 6:**
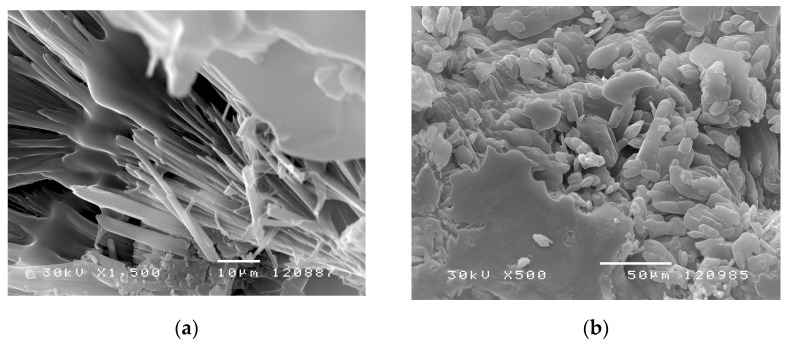
SEM micrographs of p(*N*iPAm-*co*-HPMet) sample with 1.0 mol% of EGDM: (**a**) in xerogel state (magnification 1500×, scale bar 10 μm) and (**b**) in hydrogel state 500× (magnification 500×, scale bar 50 μm).

The surface appearance of the copolymer p(*N*iPAm-*co*-HPMet) with incorporated naproxen is displayed by SEM micrographs in Figure 6a, and in the hydrogel state swollen to equilibrium in Figure 6b. Irregularly distributed naproxen crystals were observed, indicating that some part of active substance was incorporated into the channels and pores of hydrogel interior, while the other part remained on the surface.

Topology of copolymer p(*N*iPAm-*co*-HPMet) with incorporated naproxen (Figure 6a) confirms an explicit distinction in the xerogel surface appearance compared to the structure of the pure samples in the xerogel state (presented in Figure 5a,b). The surface of xerogels shows the incorporated crystals of naproxen as specific fibril structure in the bundles form into the copolymer network. Additionally, the morphology of the co-hydrogels swollen to equilibrium unambiguously points to the presence of naproxen within the polymer network (Figure 6b) in comparison to surface of pure hydrogels swollen to equilibrium (Figure 5b). It can be noticed that polymer network is quite covered with naproxen. The naproxen crystals on the surface of the hydrogel matrix are similar to the pure crystals shown in the investigation of [13,15,18,20].

### 3.6. Incorporation Efficiency of Naproxen into p(NiPAm-co-HPMet) Hydrogels

The content of incorporated naproxen into the co-hydrogels was determined from the difference in weight of the sample before and after swelling in the drug solution. The results of the incorporation efficiency of naproxen for the tested samples calculated using Equation (7) are given in Table 6.

The efficiency of naproxen incorporation into p(*N*iPAm-*co*-HPMet) is in the range 56.82–87.02%. There is a trend of decreasing efficiency of fitting as the hydrogel cross-linking density increases and the share of co-monomer HPMet increases. Synthesized porous hydrogel network allows the absorption of relatively large drug quantities.

**Figure 7:**
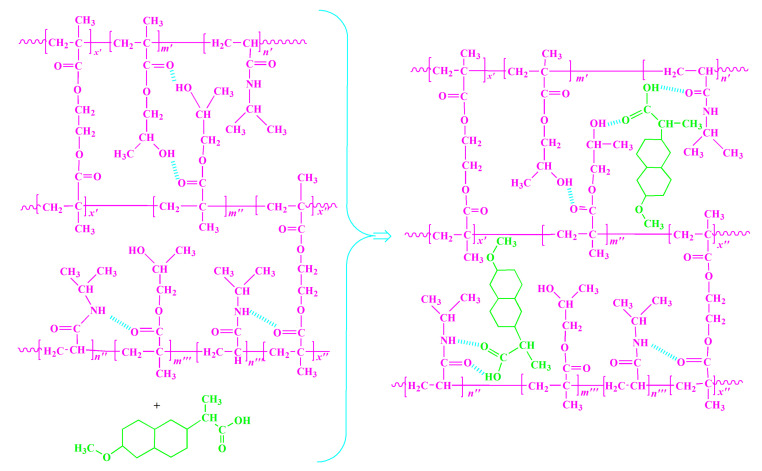
The naproxen incorporation into the p(*N*iPAm-*co*-HPMet) hydrogels.

By analyzing the results obtained by the FTIR, XRD and SEM methods, it can be concluded that naproxen was successfully incorporated into the p(*N*iPAm-*co*-HPMet) hydrogels which is schematically presented in Figure 7. Synthesized co-hydrogels are cross-linked polymer matrix and pore size provide a lot of free space in swelling state for small naproxen molecules.

### 3.7. In Vitro Thermo-Responsive Naproxen Release from p(NiPAm-co-HPMet)

**Figure 8:**
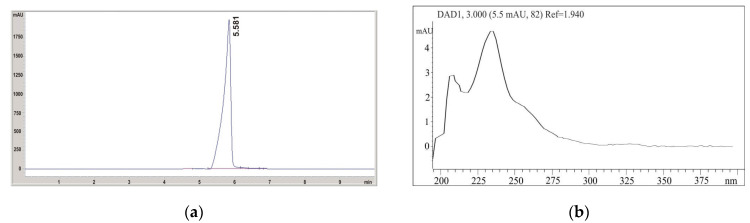
(**a**) Chromatogram of naproxen from HPLC, (**b**) UV spectrum from DAD detector.

The content of released naproxen from co-hydrogels was determined based on the Equation (8), which is valid for the linear part of the constructed calibration curve. The appearance of the peaks on the HPLC chromatogram originating from naproxen is shown in Figure 8a, and for indicated HPLC conditions it occurs at a retention time of R_t_ = 5.821 min.

The characteristic UV spectrum of naproxen (Figure 8b) shows two absorption maxima: the first is of lower intensity at λ_max_ = 206 nm and originates from the allowed π→π* transition of the disubstituted naphthalene, and the second is more intense at λ_max_ = 233 nm and originates from n→π* transitions due to the presence of the keto group, C=O, with the transition at λ_max_ = 256 nm, due to the conjugation of substituted propionic acid [62]. The limit of detection (LOD) as the lowest concentration of a substance that can be reliably (with certain statistical certainty) detected by an analytical method) is LOD = 1.19 × 10^−8^ μg/cm^3^. The limit of determination, (LOQ) as the lowest amount of a substance that can be reliably (with certain statistical certainty) determined by an analytical method, is LOQ = 3.62 × 10^−8^ μg/cm^3^ [44].

**Figure 9:**
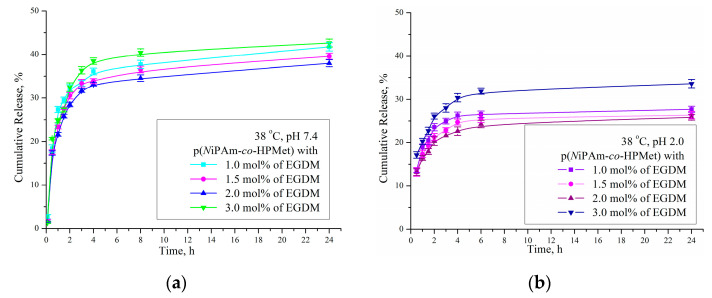
In vitro cumulative release of naproxen from p(*N*iPAm-*co*-HPMet) hydrogels at 38 °C: (**a**) at pH 7.4, (**b**) pH 2.0. In each figure, error bars represent the standard deviation of three replicates.

The results of the in vitro cumulative release of naproxen from the synthesized co-hydrogels at temperatures at 38 °C in fluid with pH 7.4 and pH 2.0, which simulate human body temperature and pH are shown in Figure 9a,b, respectively.

The results show higher values of released naproxen at a temperature above LCST. At 38 °C, in fluid pH 7.4 during 24 h p(*N*iPAM-*co*-HPMet) sample with the lowest cross-linker content (1.0 mol% EGDM) released the highest amount of naproxen (181.03 mg_naproxen_/g_xerogel_, i.e., 41.75 % of the amount incorporated). The smallest amount of naproxen was released from sample with 3 mol% of EGDM (130.36 mg_naproxen_/g_xerogel_, i.e., 42.64% of the amount of incorporated drug), in accordance with the expectation and results of the swelling. During the first 24 h about 50% of naproxen remained inside the hydrogels, and this amount would be appropriate for prolonged release.

At 38 °C in acidic fluid pH 2.0 the most naproxen was released from the co-hydrogel sample with 1.0 mol% of EGDM (119.93 mg_naproxen_/g_xerogel_, i.e., 27.69% of the incorporated amount), and the least from the sample with the highest EGDM cross-linker content (100.08 mg_naproxen_/g_xerogel_, i.e., 33.68% of the incorporated naproxen) during 24 h (Figure 9a). The amount of naproxen released depends on the composition of the hydrogel, i.e., its cross-linker content. Analysis indicates that naproxen as a drug is well stabilized by aromatic-aromatic interactions, with the greatest influence (43.2%) attributed to naphthalene-naphthalene interactions [24].

Temperature responsiveness of co-hydrogels was dominant in aggregation process and naproxen releasing. They contract and squeeze fluid with naproxen when temperature increase above VPTT. The applied amount of naproxen had not significant influence on the aggregation process. At temperatures above the LCST dominated hydrophobic interactions lead to a decrease in the hole free volume, which causes the broken intermolecular hydrogen bonds with naproxen and active drug release due to hydrogel contraction. The obtained results show a lower amount of released naproxen in acidic fluid than alkaline fluid. The reason is maybe the lower solubility of naproxen in acidic fluid. Additionally, it can be attributed to the effect of propionic acid dissociation from naproxen structure. As the molecular structure of p(*N*IPAM-*co*-HPMet) does not have an ionic functional group, low sensitivity to changes in the pH value of fluid was expected.

The kinetic parameters (*n*, *k*, and *D*) of the naproxen release mechanism from hydrogels were assessed by fitting experimental release using Equations (3)–(6) and are presented in Table 7.

Kinetic parameters of the naproxen release from the co-hydrogels show that the mechanism of fluid transport belongs to Fickian, i.e., “Less Fickian” diffusion (Table 7). The drug transport is slower than polymer chain relaxation process and is controlled by the diffusion process. Values of the diffusion coefficient D from the co-hydrogels were similar. Analysis of the in vitro thermo-responsive naproxen release shows that they could be suitable for delivering a drug for an extended period of time, more than 24 h, and up to 48 h, which would allow a more convenient application for patients. Additionally, these matrix systems show that they are particularly suitable for prolonged-release in slightly alkaline conditions (pH 7.4), e.g., in the small intestine or for topical administration. Based on the results obtained in the presented study, it was shown that formulation of thermo-sensitive p(*N*iPAm-*co*-HPMet) hydrogels with incorporated naproxen could be suitable as a carrier for modulated of NSAID release.

## 4. Conclusions

The structural analysis of the synthesized p(*N*iPAm-*co*-HPMet) copolymer by FTIR spectrum confirms the success of copolymerization process of NIPAM with HPMet comonomers. XRD difractograms and SEM micrographs verified semi-crystalline structure of obtained hydrogels. The swelling transport mechanism of the co-hydrogels at 5 °C and 38 °C in alkaline fluid corresponds to “Less Fickian” diffusion for all samples, i.e., the solvent transport in the polymeric network is considerably slower than the relaxation of polymer chains. One sample at 5 °C and pH 7.4 and all samples in acidic fluid showed anomaly in the mechanism of water transport, i.e., the swelling process is controlled by water diffusion and relaxation of polymer chains. The structural changes resulting from the incorporation of the naproxen as a model drug into the p(NiPAm-co-HPMet) copolymers determined by FTIR analysis indicate that non-covalent type intermolecular interactions between the copolymers and the incorporated naproxen were created. The surface structures of p(*N*iPAm-*co*-HPMet) in xerogels and hydrogels state, indicate the incorporation of the naproxen into the copolymer pores. The efficacy of naproxen incorporation ranged in proportion to the cross-linker content. The formulation of p(*N*iPAM-*co*-HPMet) hydrogels with incorporated naproxen in this investigation may be used as a potentially efficient drug carrier for prolonged naproxen release when body temperature increases.

## 5. Patents

Registered patent RS53220B: Ilić-Stojanović, S.; Nikolić, Lj.; Nikolić, V.; Petrović, S.D.; Stanković M. Process for synthesis of thermosensitive hydrogels and pharmaceutical applications, The Intellectual Property Office of the Republic of Serbia, http://www.zis.gov.rs/home.59.html. https://worldwide.espacenet.com/patent/search/family/046025219/publication/RS53220B?q=RS53220B.

## Figures and Tables

**Table 1:** The Fluid Diffusion Mechanism Related to the Value of Diffusion Exponent, *n.*

*n*	Fluid Transport Mechanism into Polymer Network
*n* < 0.5	Fluid penetration is considerably slower than the relaxation of polymer chains. The mechanism of fluid transport belongs to Fickian diffusion and is called “Less Fickian” diffusion.
*n* = 0.5	Fluid transport corresponds to Fickian diffusion mechanism (Case I). Diffusion degree is much lower than the polymer chain relaxation degree.
0.5 < *n* <1	Anomalous diffusion mechanism (non-Fickian diffusion) occurs when hydrogel swelling is controlled both by fluid diffusion into the matrix and polymer chain relaxation.
*n* = 1	The fluid diffusion process is much faster than the relaxation of polymer system chains (Type II, Case II).
*n* > 1	The polymer chain relaxation controls swelling, Type III (Case III, Super Case II).

**Table 2:** Equilibrium Swelling Ratio (*α*_∞_) and Kinetic Parameters (*n*, *k* and *D*) at 5 °C and 38 °C in Fluids at pH 7.4 and 2.0 for p(*N*iPAm-*co*-HPMet) Hydrogels with Different EGDM Content.

	5 °C					38 °C				
EGDM mol%	*α*_∞_	*n*	*k* × 10^2^, min^−1/2^	*D*, cm^2^/min	*R* ^2^	*α*_∞_	*n*	*k* × 10^2^, min^−1/2^	*D*, cm^2^/min	*R* ^2^
	pH 7.4					pH 7.4				
1.0	29.077	0.236	3.241	4.312 × 10^−6^	0.986	4.541	0.440	9.768	7.493 × 10^−5^	0.996
1.5	21.821	0.586	6.130	3.304 × 10^−6^	0.992	3.961	0.435	9.485	7.066 × 10^−5^	0.997
2.0	16.821	0.384	9.369	3.395 × 10^−6^	0.985	3.517	0.388	9.324	7.209 × 10^−5^	0.987
3.0	14.203	0.373	9.581	5.379 × 10^−6^	0.974	2.930	0.402	9.882	7.669 × 10^−5^	0.993
	pH 2.0					pH 2.0				

**Table 3:** The Maxima of Characteristic Absorption Bands from p(*N*iPAM-*co-*HPMet), Naproxen, p(*N*iPAM-*co-*HPMet) with Incorporated Naproxen and Value of Wavelengths Shifts after Drug Incorporation.

Wavenumber of Functional Group, (cm^−1^)			Functional Group	Shifts in Relation to the FTIR Spectra, (cm^−1^)	
p(*N*iPAm-HPMet)	Naproxen	p(*N*iPAm-*co*-HPMet) with Naproxen		p(*N*iPAm-*co*-HPMet)	Naproxen
3436		3433	ν (OH)	−3	
3311		3292	ν (NH)	−19	
	3199		ν (OH)		–
2973	2975	2975	ν_as_ (CH_3_)	+2	0
2934	2938	2938	ν_as_ (CH_2_)	+4	0
2876	2843	2878	ν_s_ (CH_3_)	+2	
1727	1728	1729	ν (C=O)	+2	+1
1652		1650	ν_s_ (C=O) amide I	−2	
1544		1552	δ (NH) amide II	+8	
	1604,1505,1481,1465	1606,1509,1458	ν (C=C) Ar		+2, +4, –, −7
1461		1465	ν(C-C)	+4	
	1418		ν(C-O)		
1387		1389	δ(CH)-isopropyl	+2	
	1394, 1264	1389, 1265	δ(OH)		−5, +1
1240	1227	1230	ν_as_(C-O)	10	+3
1173	1176	1174	ν_as_(C-O)	+1	−2
	1157	1159	ν(C-O) + δ(CH)Ar		+2
1131	1028	1123	ν_s_ (C-O)	−8	−5
927	925	925	γ (OH)	−2	0
883	896, 864, 819	895, 855, 819	γ (C-H) Ar	+12	+1, −9,0
664	674	675	γ (OH)	+1	+1

**Table 4:** The Values of Diffraction Angles and the Diffraction Peaks Intensities for the Co-Hydrogel Samples before and after Naproxen Incorporation.

Hydrogel Sample	Increment 7–34° (*2Θ*)			
	*2Θ* (°)		Intensity (*Arb. Units*) ^a^	
	I Peak(s)	II Peak (f)	I_f_	I_s_
p(*N*iPAm-*co*-HPMet) 1 mol% EGDM	8.2	20.0	500	530
p(*N*iPAm-*co*-HPMet) 2 mol% EGDM	8.5	19.0	540	500
p(*N*iPAm-*co*-HPMet) 3 mol% EGDM	8.5	19.8	540	430
p(*N*iPAm-*co*-HPMet) with incorporated naproxen	8.4	20.8	550	400
p(*N*iPAm)	8.2	20.0	500	350

^a^ (arbitrary units).

**Table 5:** The Values of The X-ray Crystallinity Index, CrI, and the Variations in the Crystallinity index, Δ(CrI) of Co-Hydrogels before and after Naproxen Incorporation.

Hydrogel Sample	CrI ^a^	(ΔCrI), % ^a^
p(*N*iPAm-*co*-HPMet) 1 mol% EGDM	−6.000 ± 0.07	−120.000 ± 0.08
p(*N*iPAm-*co*-HPMet) 2 mol% EGDM	7.407 ± 0.04	−75.308 ± 0.05
p(*N*iPAm-*co*-HPMet) 3 mol% EGDM	20.370 ± 0.09	−32.099 ± 0.03
p(*N*iPAm-*co*-HPMet) with incorporated naproxen	27.273 ± 0.02	−9.091 ± 0.04
p(*N*iPAm)	30.000 ± 0.03	–

^a^ All data are presented as the average ± standard deviation of three replicates.

**Table 6:** The Mass of Incorporated Naproxen (*L*_g_) into the Co-Hydrogels and Naproxen Incorporation Efficiency (*η*_naproxen_).

p(*N*iPAm-*co*-HPMet) with	*L*_g_ Naproxen (mg/g_xerogel_) ^a^	*η*_naproxen_ (%) ^a^
1.0 mol% of EGDM	433.6 ± 0.14	86.72 ± 0.12
1.5 mol% of EGDM	421.7 ± 0.12	84.34 ± 0.09
2.0 mol% of EGDM	408.2 ± 0.09	81.64 ± 0.08
3.0 mol% of EGDM	312.8 ± 0.16	62.56 ± 0.11

^a^ All data are presented as the average ± standard deviation of three replicates.

**Table 7:** Kinetic Parameters (*n*, *k* and *D*) of Diffusion for Released Naproxen from p(*N*iPAm-*co*-HPMet) Hydrogels at 38 °C at pH 2.0 and pH 7.4.

EGDM, mol%	mg_naproxen_/g_xerogel_^a^	*n*	*k* (min^−1/2^)	*R* ^2^	*D* (cm^2^/min)	*%* ^a^
**pH 7.4**						
1.0	181.03 ± 0.07	0.332	0.676	0.952	3.59 × 10^−3^	41.75 ± 0.07
1.5	166.74 ± 0.06	0.367	0.647	0.988	3.29 × 10^−3^	39.61 ± 0.09
2.0	155.01 ± 0.01	0.346	0.651	0.994	3.33 × 10^−3^	37.99 ± 0.06
3.0	133.37 ± 0.03	0.321	0.605	0.976	2.87 × 10^−3^	42.64 ± 0.07
**pH 2.0**						
1.0	119.93 ± 0.02	0.340	0.649	0.955	3.31 × 10^−3^	27.69 ± 0.04
1.5	113.28 ± 0.06	0.311	0.639	0.981	3.20 × 10^−3^	26.86 ± 0.07
2.0	108.59 ± 0.04	0.283	0.627	0.987	3.09 × 10^−3^	26.66 ± 0.03
3.0	100.08 ± 0.03	0.285	0.615	0.984	2.97 × 10^−3^	33.68 ± 0.02

^a^ All data are presented as the average ± standard deviation of three replicates.

## Data Availability

No new data were created or analyzed in this study.
